# Knockdown of lncRNA HAGLROS inhibits metastasis and promotes apoptosis in nephroblastoma cells by inhibition of autophagy

**DOI:** 10.1080/21655979.2021.2023984

**Published:** 2022-03-31

**Authors:** Pugui Li, Kun Zhang, Shijie Tang, Weizhu Tang

**Affiliations:** a Department of General Surgery, Taikang Xianlin Drum Tower Hospital, Nanjing, China; b Department of Plastic Surgery and Burn Center, Second Affiliated Hospital, Shantou University Medical College, Shantou, China; c Department of Urology, Nanping First Hospital, Nanping, China

**Keywords:** Nephroblastoma, HAGLROS, autophagy, metastasis, apoptosis

## Abstract

Nephroblastoma, or Wilms tumor, is a primary renal malignant tumor that easily occurs in children. Previous studies have revealed the regulatory functions of LncRNA in nephroblastoma. LncRNA HAGLROS functions as a tumor promotor in various cancers including hepatocellular carcinoma, ovarian cancer and colorectal cancer. In this study, the HAGLROS expression in nephroblastoma cells was assayed through qRT-PCR. Cell proliferation assessment employed CCK-8. Moreover, the migration and invasion of cells were examined separately through wound healing and transwell assay. Moreover, flow cytometric analysis and Western blot assay were applied to evaluate cell apoptosis. Rapamycin and 3-methyladenine were used to serve as autophagy activator or inhibitor, respectively. In addition, autophagy was identified by immunofluorescence staining and Western blot analysis. Experiment results showed that HAGLROS expressed highly in nephroblastoma cell lines. HAGLROS knockdown prevented cells from proliferating, and also showed suppressive impact on migration and invasion in HFWT cells. In addition, knockdown of HAGLROS showed a facilitative effect on apoptosis and an inhibitory effect on autophagy. Stimulation of autophagy alleviated HAGLROS silencing-induced apoptosis, while inhibition of autophagy reversed the effect in nephroblastoma cells. In summary, our results revealed that HAGLROS executed an oncogenic role in the progress of nephroblastoma, offering a new perspective on the strategy for nephroblastoma therapy.

## Introduction

Nephroblastoma, or Wilms tumor, is a primary malignant tumor that occurs in children [1–3]. It accounts for 6–7% of malignant tumors in children younger than 15, about 75% of which are distributed in children under 5 years old [4]. Treatment for Wilms’ tumor is administered according to its diagnosis and stage, usually surgical resection, chemotherapy, or targeted therapy are implemented [5]. Among these approach, comprehensive treatment based on the combination of surgery, chemotherapy and radiotherapy is usually a more practical treatment for all stages of nephroblastoma [6]. Although the total survival rate is over 90% at 5 years, plenty of side effects and sequelae of drug treatment such as malnutrition, cardiac dysfunction and secondary malignancies, still trouble the patients [7]. In addition, there are still a few of children died of relapse, metastasis and resistance to chemotherapy and other reasons [8]. Thus, great efforts on exploring the pathogenesis of nephroblastoma may throw light on the identification of molecular diagnostic markers or therapeutic targets for nephroblastoma.

Long non-coding RNAs (lncRNAs) are a class of RNAs that have no protein coding capacity with a length of over 200 nucleotides [9]. LncRNAs are capable of regulating the transcription, translation, and protein modification by interacting with DNAs, RNAs or proteins [10]. It is widely accepted that LncRNAs are intimately involved in the formation, development and metastasis of numerous cancers [11,12]. Earlier researches indicated that several LncRNAs are critical in cell proliferation, migration, apoptosis and cell cycle of nephroblastoma [13,14]. Based on the study of Ge et al., lncRNA deleted in lymphocytic leukemia 1 (DLEU1) expression was upregulated in nephroblastoma tissues and cell lines and knockdown of DLEU1 suppressed the proliferation, migration and invasion of GHINK-1 cells via targeting the miR-300/HOXC8 axis [15]. HAGLROS, one of the lncRNAs, has been reported to be abnormally expressed in many types of malignancies, presenting a carcinogenic role and contributing to the malignant processes of a number of cancers [16,17]. For instance, Wei et al. reported that HAGLROS promoted cell proliferation and cell autophagy but inhibited apoptosis through mediating miR-5095/ATG12 axis in hepatocellular carcinoma cells [18]. In addition, Wang et al. demonstrated that HAGLROS expression was correlated with tumor stage and lymphatic metastasis of lung cancer. Furthermore, HAGLROS accelerated proliferation and migration of Lung cancer cells by regulating microRNA-152 [19]. However, the biological role of HAGLROS in the occurrence and progression of nephroblastoma remains incompletely discovered.

This study was designed to look into the expression and biological role of lncRNA HAGLROS in nephroblastoma cells. We demonstrated that HAGLROS expression was higher in nephroblastoma cell lines and knockdown of HAGLROS yielded suppressive effects on the development of Wilms tumor, which provides a reliable new target for the therapy of nephroblastoma. We hypothesized that HAGLROS silencing inhibits nephroblastoma development by regulating cell proliferation, migration, invasion and apoptosis. In addition, we intend to explore whether HAGLROS silencing promotes nephroblastoma cell apoptosis by the inhibition of autophagy.

## Materials and methods

### Cell culture and treatment

Normal human embryonic kidney cell line HEK-293 T and nephroblastoma cell lines HFWT, 17–94 and WiT49 were supplied by American Type Culture Collection (ATCC; Manassas, VA, USA). For the culture of these cells, they were usually conducted in Dulbecco’s Modified Eagle Medium (DMEM; Invitrogen, Carlsbad, CA, USA) 10% fetal bovine serum (FBS) and 1% penicillin/streptomycin. The temperature of the incubator was controlled at 37°C and the CO_2_ concentration was 5%. For the induction and inhibition of autophagy, cells were subjected to treatment separately with 0.2 μg/ml rapamycin (an autophagy activator; Sigma, St Louis, MO, USA) or 5 mM 3-methyladenine (3 M, an autophagy inhibitor; Sigma) for 24 h [20,21].

### Cell transfection

The short-hairpin RNA against HAGLROS (shRNA-HAGLROS-1/2) and negative control (shRNA-NC) were constructed by GenePharma (Shanghai, China). HFWT cells were seeded in six-well plates and transfected with these shRNAs with the help of Lipofectamine 2000 Reagent (Life Technologies Corporation, Grand Island, NY, USA) in line with the standard procedures of the vendor. 48 h later, cells were collected to make subsequent experiment studies [22].

### Cell proliferation

Cell proliferation assessment was undertaken with the application of Cell counting kit-8 (CCK-8) assay [23]. After transfection with shRNA-HAGLROS-1 or shRNA-NC, HFWT cell line was cultured in 96-well plates at a density of 5 × 10^3^ cells/well for various time periods (24 h, 48 h and 72 h). Then CCK-8 reagent was incubated with the plates seeded with cells at 37°C in 5% CO_2_ for 2 h. The measurement of optical density values employed a microplate reader (RT-3001; Thermo Fisher Scientific, Waltham, MA, USA) under 450 nm wavelength.

### Quantitative real-time polymerase chain reaction (qRT-PCR)

Extraction of total RNA from nephroblastoma cell lines and transfected cells employed TRIzol reagent (Invitrogen) and DNase I was used to remove genomic contamination. A reverse transcription for cDNA synthesis was performed by PrimeScript RT Master Mix (Takara Biomedical Technology, Beijing, China). The PCR reaction was undertaken making use of the SYBR Premix Ex Taq™ II kit (Takara, Shiga, Japan) at an ABI PRISM 7000 Sequence Detection System (Applied Biosystems, Foster City, CA, USA). The primer sequences for PCR are presented as below: HAGLROS: 5′-TGTCACCCTTAAATACCGCTCT-3′ (forward) and 5′-CTTCCTCCCACACAAATACTCC-3′ (reverse); GAPDH: 5′-GGGAAACTGTGGCGTGAT-3′ (forward) and 5′-GAGTGGGTGTCGCTGTTGA-3′ (reverse). GAPDH was worked as the control of HAGLROS and the results were measured by using the 2^−ΔΔCt^ method [24].

### Wound healing assay

After transfection, HFWT cells were grown in 6-well plates at a density of 4 × 10^5^ cells /well until 90% confluence. The cell monolayer was scratched in a straight line utilizing a white pipette tip. Washed three times using serum-free medium, the monolayer was cultured for 24 h and the area occupied by migrated cells in the scratch was evaluated by an inverted microscopy [25].

### Transwell assay

The experiment of cell invasive ability of transfected HFWT cells was undertaken with the application of transwell chamber (Corning Costar, Cambridge, MA) [26]. Firstly, 0.1 mL of Matrigel was used to coat the chambers at 37°C for 1 h. After transfection, HFWT cells were suspended in DMEM without serum and placed in the upper wells. Meanwhile, the lower chamber was loaded with DMEM with 10% FBS. 24 h later, a swab was adopted to wipe off the non-migrated cells on the transwell membrane gently. Then the cells crossing the membrane were fixed, stained with 0.05% crystal violet and counted by a microscope under in five randomly chosen fields.

### Cell apoptosis analysis

The treatment of HFWT cells with 0.2 μg/ml rapamycin or 5 mM 3 M was conducted for 24 h after transfected with shRNA-HAGLROS-1 or shRNA-NC. Treated differently, the cultured cells were harvested, rinsed twice with precooled PBS and resuspended in binding buffer. Then, the cells were double-labeled with Annexin V-FITC and PI apoptosis detection kits (Ribobio, Guangzhou, China) in keeping with the supplier’s guidance. Cell apoptosis was examined by means of BD LSRII Flow Cytometer System (BD Biosciences) and analyzed by Flowjo Software (Tree Star, Ashland, OR, USA) [27].

### Immunofluorescence staining

The fixation and permeabilization of cell sections were performed with paraformaldehyde (4%) and Triton X-100 (0.1%), respectively. Then, 5% skimmed milk was added in 0.01 M PBS for blocking the cells which were incubated with primary antibodies targeting LC3B (1 µg/ml; Abcam) overnight at 4°C. Twice-washed with PBS, the cells were subsequently cultured with Goat Anti-Rabbit IgG H&L secondary antibody (1:500; Abcam). The coverslips were then rinsed three times with PBS and visualized with the aid of a fluorescence microscope (Olympus, Tokyo, Japan) [28].

### Western blot analysis

Total protein was extracted from different treated cells using RIP assay lysis buffer (Beyotime Biotechnology, Shanghai, China) supplemented with protease inhibitor and the protein concentration was measured by BCA Protein Assay Kit (Pierce, Appleton, WI). Then, a total of 30 μg protein per well were separated by 10% SDS-PAGE and transferring onto a PVDF membrane (Millipore, Billerica, MA), and the separated membrane was blocked by the use of 5% nonfat milk solution for 1 h followed by an overnight incubation with primary antibodies against Bcl-2, Bax, cleaved caspase3, LC3B, ATG7, Beclin1 and GAPDH (1:1000, Santa Cruz, USA) at 4°C. At the end of three washes, the membrane was subjected to incubation for another 1 h using horseradish peroxidase-conjugated secondary antibody. Visualization of the bands was carried out using the enhanced chemiluminescence (ECL) system (Beyotime, Shanghai, China) and quantification using Image J (NIH, USA) [29].

### Statistical analysis

All data analysis was implemented through SPSS 19.0 (Chicago, IL, USA). An unpaired two-tailed Student’s t-test was applied for the comparison of differences between two groups. Differences among multiple groups were analyzed using one-way ANOVA with a post hoc Bonferroni multiple comparison test. The experiment results were recorded in the way of mean ± SD. A statistical significance was identified when the P value was <0.05.

## Results

In this study, we investigated the biological roles of HAGLROS and the potential mechanism in nephroblastoma HFWT cells. The data showed that the silencing of HAGLROS suppressed HFWT cellproliferation, migration and invasion. In addition, HAGLROS silencing repressed autophagy and induced cell apoptosis. Moreover, activation of autophagy inhibited HAGLROS silencing-mediated increased cell apoptosis, while the inhibition of autophagy promoted the apoptosis of HFWT cells.

### HAGLROS expression is upregulated in nephroblastoma cell lines

**Figure 1.**
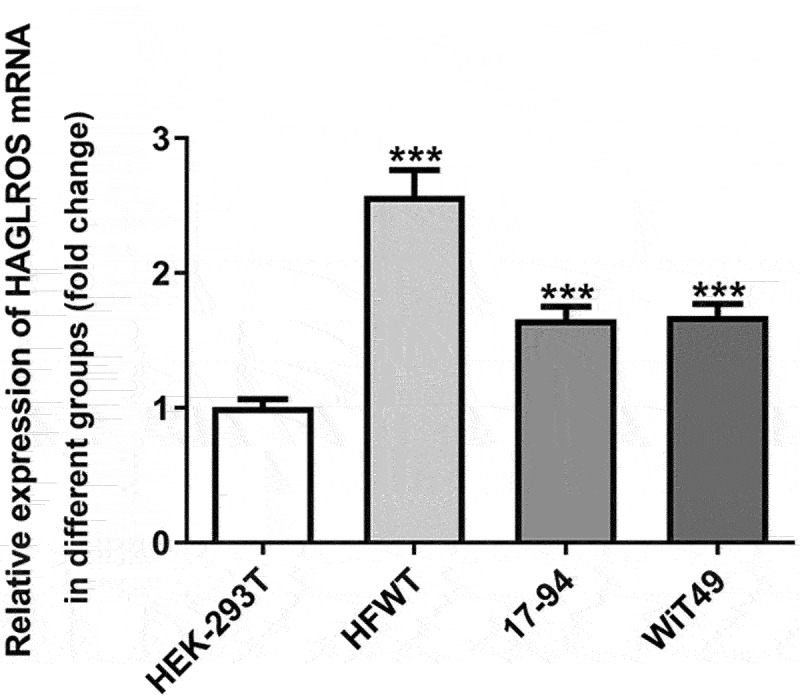
HAGLROS expression is upregulated in several nephroblastoma cell lines. qRT-PCR was performed to detect HAGLROS mRNA expression in nephroblastoma cells and control cell line HEK-293 T. ***P < 0.001 vs HEK-293 T.

To examine the function of lncRNA HAGLROS in nephroblastoma, the detection of HAGLROS expression in several nephroblastoma cell lines and normal human embryonic kidney cells employed qRT-PCR. As shown in Figure 1, HAGLROS mRNA expression in nephroblastoma cells was remarkably higher (vs Control). The results suggest that HAGLROS might be associated with progression of nephroblastoma.

### Knockdown of HAGLROS suppressed the proliferation, migration and invasion of HFWT cells

**Figure 2.**
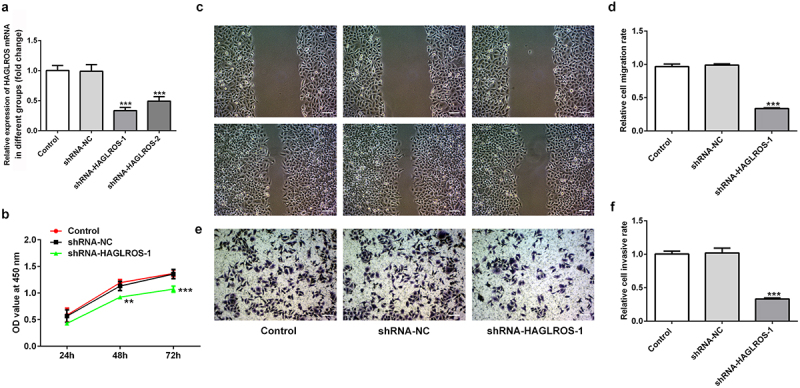
Effects of HAGLROS knockdown on the proliferation, migration and invasion of HFWT cells. (a) mRNA expression of HAGLROS in HFWT cells was evaluated adopting qRT-PCR. (b) Cell proliferation was assessed by CCK-8 assay after transfection with shRNA-HAGLROS-1 or shRNA-NC. (c-d) Cell migration detection of HFWT cells with different treatments employed wound healing assay. Scale bar: 100 μm. (e-f) Transwell experiment was applied for the identification of cell invasion of HFWT cells with different treatments. Scale bar: 100 μm. **P < 0.01, ***P < 0.001 vs control.

For the exploration of the influence of HAGLROS on nephroblastoma cells, HAGLROS expression in HFWT cells after its knockdown was measured via qRT-PCR (Figure 2(a)). shRNA-HAGLROS-1 showed the better transfetion and was chosen to perform follow-up experiments. Next, we found that HAGLROS silencing inhibited HFWT cell proliferation in comparison with the control and negative control groups (Figure 2(b)). In addition, the migration and invasion capacity of HFWT cells were evidently repressed by transfection with shRNA-HAGLROS-1 (Figure 2 (c-f)). These data indicate that HAGLROS silencing exerts suppressive roles in proliferating, migrating and invading of nephroblastoma cells.

### HAGLROS silencing promoted cell apoptosis in HFWT cells

**Figure 3.**
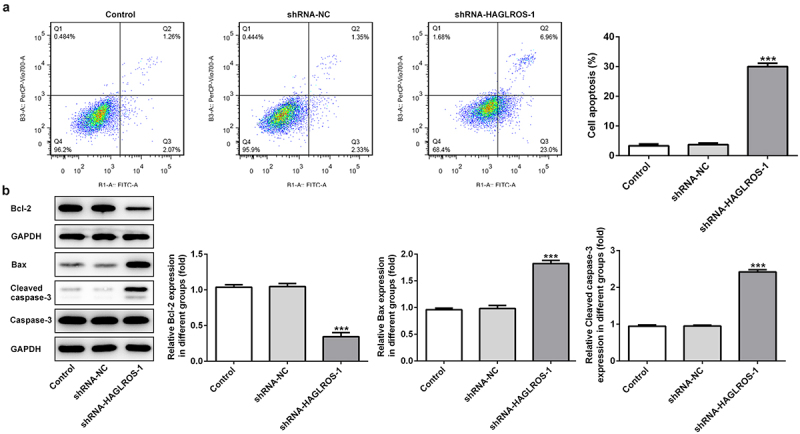
Effects of HAGLROS knockdown on the apoptosis of HFWT cells. (a) Flow cytometry was undertaken for the analysis of the apoptosis rate in transfected cells. (b) Western blot was implemented to measure the protein levels of Bcl-2, Bax and cleaved caspase 3 in HFWT cells transfected with shRNA-HAGLROS-1 or shRNA-NC. ***P < 0.001 versus control.

To further study the effects of HAGLROS on nephroblastoma, cell apoptosis detection was undertaken. As indicated in Figure 3(a), flow cytometric analysis revealed that downregulation of HAGLROS greatly increased cell apoptosis rate by contrast to the control and negative control groups. Moreover, the results from Western blot analysis showed that Bcl-2 level was reduced but the levels of Bax and cleaved caspase 3 were significantly increased in shRNA-HAGLROS-1 group when compared with the controls (Figure 3(b)). The data indicate that HAGLROS plays a regulatory role in cell apoptosis of nephroblastoma.

### HAGLROS silencing inhibits the autophagy in nephroblastoma cells

**Figure 4.**
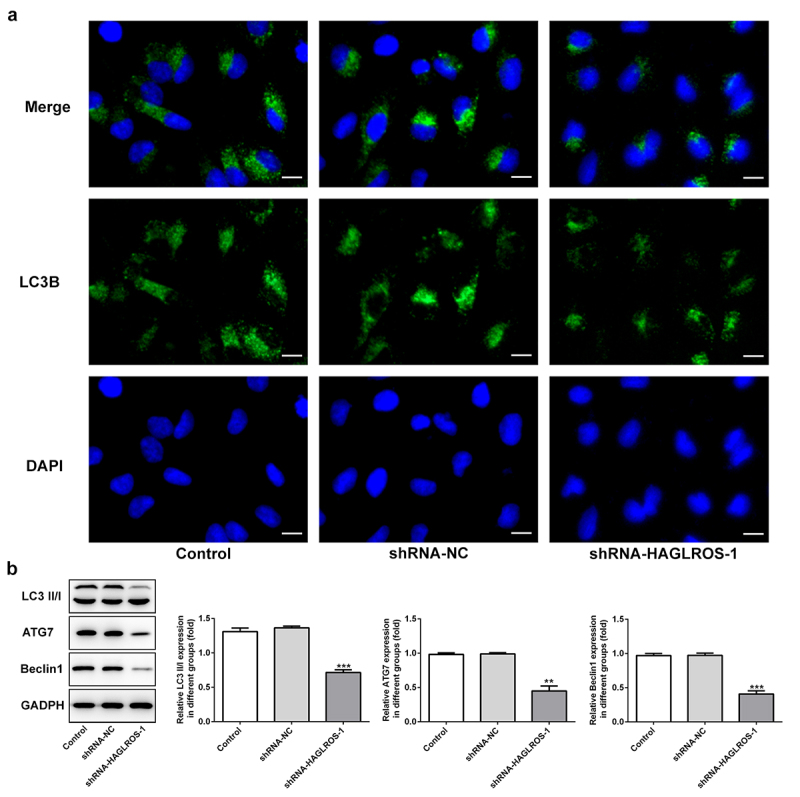
Impacts of HAGLROS silencing on the autophagy of HFWT cells. (a) Immunofluorescence staining was applied for exploring the level of LC3II in cells transfected with shRNA-HAGLROS-1 or shRNA-NC. Scale bar: 50 μm. (b) Protein levels of LC3II/I, ATG7 and Beclin1 in HAGLROS silenced cells by Western blot assay. **P < 0.01, ***P < 0.001 versus control.

The objective of this set of experiments was to identify the effect of HAGLROS on cell autophagy in HFWT cells. The results obtained from immunofluorescence staining showed that LC3II level was remarkably decreased in cells transfected with shRNA-HAGLROS-1 compared with transfection with shRNA-NC (Figure 4(a)). Furthermore, HAGLROS silencing markedly enhanced the protein levels of LC3II/I, ATG7 and Beclin1 according to Western blot assay (Figure 4(b)). Thus, HAGLROS may be involved in nephroblastoma cell autophagy.

### Effects of cell autophagy on the apoptosis of HFWT cells with HAGLROS silencing

**Figure 5.**
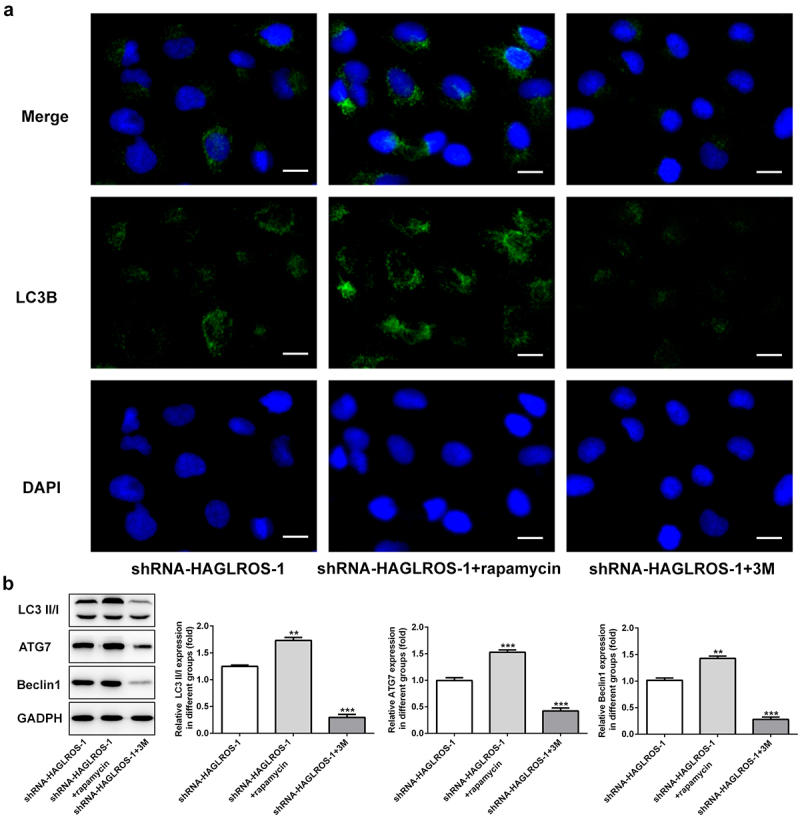
The autophagy in HFWT cells after transfection with shRNA-HAGLROS-1.(a) Level of LC3II in cells transfected with shRNA-HAGLROS-1 with rapamycin or 3 M was examined by immunofluorescence staining. Scale bar: 50 μm. (b) Western blot was employed to calculate the levels of LC3II/I, ATG7 and Beclin1 in HAGLROS silenced cells with rapamycin or 3 M. **P < 0.01, ***P < 0.001 versus control.

**Figure 6.**
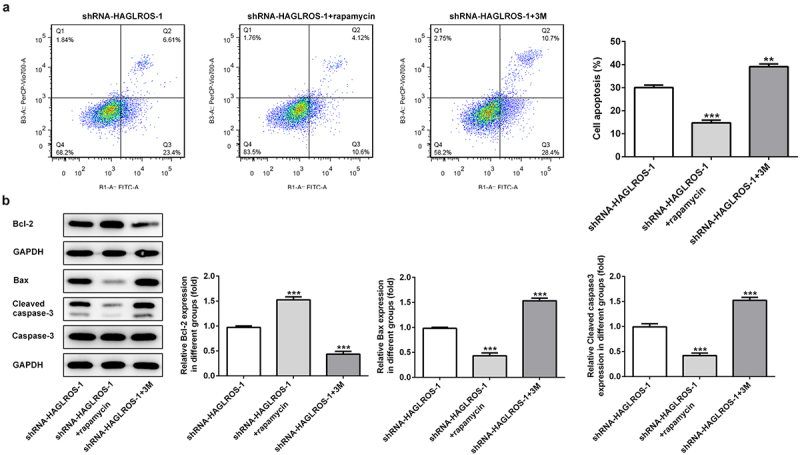
The regulatory effects of autophagy on apoptosis in HFWT cells after transfection with shRNA-HAGLROS-1. (a) Flow cytometry was implemented to determine the apoptosis rate in transfected cells with rapamycin or 3 M. (b) Western blot was executed to elucidate the levels of Bcl-2, Bax and cleaved caspase 3 in HFWT cells transfected with shRNA-HAGLROS-1 with rapamycin or 3 M. **P < 0.01, ***P < 0.001 versus control.

Finally, we elucidated the relationship between autophagy and apoptosis in HFWT cells after transfection with shRNA-HAGLROS-1. As shown in Figure 5(a), rapamycin significantly increased the protein level of LC3II while 3 M reduced the LC3II level in HFWT cells transfected with shRNA-HAGLROS-1. Consistently, the protein levels of LC3II/I, ATG7 and Beclin1 were conspicuously elevated after treatment of rapamycin while 3 M reduced the protein levels (Figure 5(b)), indicating that autophagy can be regulated by rapamycin or 3 M in in HFWT cells. On the basis of flow cytometric analysis, activation of autophagy reduced cell apoptosis rate while inhibition of autophagy facilitated the apoptosis upon transfection with shRNA-HAGLROS-1 (Figure 6(a)). Moreover, rapamycin increased the Bcl-2 level and attenuated the activities of Bax and cleaved caspase 3. However, 3 M showed opposite effects on in HFWT cells after transfection with shRNA-HAGLROS-1 (Figure 6(b)). These results suggest that autophagy restrains apoptosis while autophagy inhibition promotes apoptosis after HAGLROS silencing.

## Discussion

Nephroblastoma is one of the most common cancers with a significant direct impact on the survival and health for children [30]. The molecular mechanisms underlying nephroblastoma are usually found to be linked with noncoding RNAs, especially lncRNAs [31,32]. Nevertheless, the key lncRNAs that take part in the occurrence and development of nephroblastoma are still incomplete grabbed. It was found in our study that HAGLROS expressed higher in nephroblastoma cell lines. HAGLROS silencing suppressed the proliferation of nephroblastoma cell, repressed migration, invasion and autophagy, but promoted apoptosis. Moreover, a negative regulation between apoptosis and autophagy was elucidated in HFWT cells after HAGLROS was downregulated.

Multiple evidences have confirmed that many lncRNAs play crucial roles in cell biological activities including proliferation, migration and invasion in nephroblastoma [23,33]. Zhang et al. reported that SOX21-AS1 expression was markedly elevated in the tissues and cell lines of nephroblastoma. SOX21-AS1 knockdown diminished the proliferation and colony-forming ability of nephroblastoma cells and inhibited cell cycle [34]. Another study showed that SNHG6 was found to be overexpressed in nephroblastoma tissues and knockdown of SNHG6 suppressed cell proliferation, migration and incursion via elevating miR-15a expression [35]. Consistently, our study revealed that HAGLROS expression was increased in nephroblastoma cells, among which HFWT cells showed the highest HAGLROS level. In addition, we found that the proliferation, migration and invasion of HFWT cells were inhibited after downregulation of HAGLROS, suggesting the oncogenic role of HAGLROS in nephroblastoma. HAGLROS was also found to affect proliferation, migration and invasion in other types of cancers. Wei et al. revealed that HAGLROS level was significantly higher in hepatocellular carcinoma (HCC) tissues and was correlated with clinical characteristics, tumor stage or tumor differentiation of patients with HCC [18]. Chen et al. showed that HAGLROS expression was significantly elevated and related to outcomes of gastric cancer (GC) patients. Knockdown of HAGLROS considerably inhibited the cell proliferation, invasion and migration by regulation of mTOR signals [36].

Numerous studies conducted on apoptosis in recent years have demonstrated its role in the development of disease. Nevertheless, apoptosis is not the only factor that determines the destiny of a cell [37]. Autophagy has been shown to engage in complex interplay with apoptosis and proved to contribute to cell survival or death according to the different cell microenvironment [38]. The role of the double-edged sword autophagy modulation in cancers is researching continually and excessive autophagy can lead to cell death [39]. One study showed that inhibition of autophagy reduced cell activity and induced cell apoptosis in childhood nephroblastoma [40]. In our study, HAGLROS silencing induced cell apoptosis by reducing cell apoptosis rate, inhibited Bcl-2 level and enhanced the activities of Bax and cleaved caspase 3 in nephroblastoma cell line HFWT. Furthermore, we also found that downregulation of HAGLROS suppressed autophagy through inhibiting the expressions of autophagy-related proteins, including ATG7, Beclin1 and LC3II/I. For the purpose of studying the impacts of autophagy on cell apoptosis in nephroblastoma, we regulated autophagy level in HFWT cells by administration of rapamycin or 3 M. The results revealed that activating autophagy by rapamycin significantly repressed cell apoptosis while inhibition of autophagy by 3 M increased the rate of cell apoptosis. Similarly, Western blot assay showed an increased expression of Bcl-2 and reduced levels of Bax and cleaved caspase 3 after induction of autophagy whereas 3 M treatment caused the opposite phenomenon inversely, which was in line with previous results that lncRNA HAGLROS facilitated cell proliferation, suppressed apoptosis and accelerated autophagy in hepatocellular carcinoma cells through the regulation of miR-5095/ATG12 axis [18]. However, we did not perform animal experiments or examine HAGLROS expression in clinical samples in this study. In addition, we only focus on the biological functions of HAGLROS in one nephroblastoma cell line HFWT. Thus, we plan to explore roles of HAGLROS in other nephroblastoma cell lines to verify our results and perform animal and clinical trials about HAGLROS in the further experiments. In addition, we will explore the expression pattern of HAGLROS in nephroblastoma by bioinformatics analysis in further study.

## Conclusion

In summary, our findings revealed that HAGLROS expression might be associated with pathogenesis of nephroblastoma. Knockdown of HAGLROS suppressed cell proliferation and metastasis, accelerated apoptosis and diminished autophagy. HAGLROS may regulate nephroblastoma cell apoptosis via mediating the activation of autophagy. Our study provides a new strategy for the targeted therapy of nephroblastoma.

## Highlights

- HAGLROS is highly expressed in nephroblastoma cell lines.
- The silencing of HAGLROS inhibits the proliferation, migration and invasion of HFWT cells.
- Downregulated HAGLROS promotes HFWT cell apopto sis and suppresses HFWT autophagy.
- Activation of autophagy reduces HAGLROS-mediated cell apoptosis, while autophagy inhibition shows the opposite effects on HFWT cells.
